# Trends of incidence and treatment strategies for operatively treated distal fibula fractures from 2005 to 2019: a nationwide register analysis

**DOI:** 10.1007/s00402-021-04232-0

**Published:** 2021-11-07

**Authors:** Alexander Milstrey, Sebastian Felix Baumbach, Alexander Pfleiderer, Julia Evers, Wolfgang Boecker, Michael J Raschke, Hans Polzer, Sabine Ochman

**Affiliations:** 1 Department of Trauma-, Hand- and Reconstructive Surgery, University Hospital Muenster, WWU Muenster, Waldeyer Street 1, 48149 Muenster, Germany; 2 Department of Orthopaedics and Trauma Surgery, Musculosceletal University Center Munich (MUM), University Hospital, LMU Munich, Munich, Germany

**Keywords:** Ankle fracture, Distal fibula fracture, Epidemiology, Treatment strategy, Locking plate, Fibula nail

## Abstract

**Introduction:**

Valid epidemiological data about distal fibular fractures and their treatment strategies are missing. Innovative osteosynthesis techniques were introduced and improved during the past 15 years. The aim of this study was to investigate the epidemiologic development and the implementation of new treatment strategies in a nationwide register in Germany over a period of 15 years.

**Materials and methods:**

Data of the German Federal Statistical Office from 2005 until 2019 were screened. Adults with a fracture of the distal fibula were included. Data were separated for gender, age and treatment strategy.

**Results:**

During the past 15 years, there was a steady annual incidence of distal fibula fractures of 74 ± 32 per 100,000 people without any significant changes (*p* = 0.436). 60.1% ± 0.6% of all fractures occurred in females. The annual incidence for male was nearly constant over the different age groups, whereas for female, there was a clear increase in incidence above the age of 40. Whereas 66% of fractures in between 20 and 30 years of age occurred in male, approximately 70% of fractures above the age of 60 occurred in females. The relative quantity of locking plates increased from 2% in 2005 to 34% in 2019. In 2019, only 1.02% of the patients were operated with an intramedullary nail.

**Conclusions:**

Operatively treated distal fibular fractures revealed an age dependent increase in incidence in postmenopausal women compared to younger females. Regarding the treatment strategy, there was an increase in application of locking plates. The data implicate a typical fragility fracture related age and gender distribution for distal fibula fractures.

**Supplementary Information:**

The online version contains supplementary material available at 10.1007/s00402-021-04232-0.

## Introduction

Ankle fractures are among the most common fractures in adult people [1]. There is a major research focus on gaining inside into the individual and general factors associated to this injury. This is the prerequisite to enhance the treatment quality, leading to a better outcome and a reduction of the socioeconomic burden. National registries enable researchers not only to analyze the incidence of ankle fractures, but also to monitor the implementation of novel treatment strategies in daily practice.

Reported incidence rates range from 42 to 187/100,000 people per year [2–7], increasing to 300/100,000 people per year in the elderly population. [8–10] But, the majority of these studies are based on data of single centers and are, therefore, limited by a selection bias and by sample size. Data from national registries overcome both of these limitations [11]. To our best knowledge, Thur et al*.* [5] published the only long-term national register study on ankle fractures. The reported annual ankle fracture incidence in Sweden was 71/100,000 people per year without significant alterations over the observed study period. However, the data presented covered a rather historic period from 1987 to 2004. Therefore, we are missing current and valid data on the incidence of ankle fractures.

Thur et al*.* [5] did also not report on the frequencies of different treatment strategies and their development over time. In the last two decades, several new exciting treatment strategies have been developed. These include anatomical locking plates [12] and intramedullary nails[13] for distal fibular fractures. Locking plates and intramedullary nails were designed predominantly for the elderly population, as they were shown to enhance the biomechanical stability and reduce wound healing problems especially in patients at risk [14–19]. However, the authors are not aware of any study evaluating the actual implementation of these modern treatment strategies into daily practice.

The aim of the present study was, therefore, to evaluate the incidence and treatment strategies for operatively treated ankle fractures with a fracture to the lateral malleolus in the most populous country in Europe over a 15-year period based on national registry data.

## Materials and methods

The herein presented study is a longitudinal national registry study based on anonymized data sets. Therefore, the study needed no approval of the local ethics committee.

### National registry

In Germany, any in-house medical procedure must be reported to the German Federal Statistical Office to receive payment for the reported case [20]. Therefore, every German hospital contributes to the national registry and every in-house procedure is recorded. Data are transmitted anonymized and coded based on the German procedure classification system (OPS). The annual data sets can be purchased publicly through the German Federal Statistical Office and form the basis for the official annual national health care report. Each annual data set provides the OPS codes separated by age in 5-year increments and gender (male, female). For the current study, the data sets of the past 15 years (2005–2019) were purchased, comprising of a total amount of 266,911,590 procedures.

### Data analysis

The aim was to identify all procedures related to distal fibula fractures in adult patients (≥ 20 years). Therefore, the data set was screened for the following OPS codes: 5–790.3r, 5–790.4r, 5–790.4r, 5–793.3r, 5–793.ar, 5–793.br, 5–793.cr, 5–793.kr, 5–794.2,, 5–794.ar, 5–794.br, 5–794.cr, 5–794.gr, 5–794.kr (Supp. 1).

The resulting 15 individual data sets were merged, and the age groups recalculated to 10-year increments (20–29 years, 30–39 years, 40–49 years, etc.). The annual incidence was calculated as the sum of all procedures per year (sum out of all OPS codes) divided by the year- and age-matched German population. Annual population data were also retrieved from the German Federal Statistical Office [21]

The second aim of this study was to evaluate the treatment trends over the past two decades for anatomical locking plates and intramedullary nails. Therefore, the OPS codes and incidences were grouped according to these treatment strategies: Conventional plates (OPS codes: 5–793.3r, 5–794.2), angular stable plates (OPS codes: 5–793.kr, 5–794.kr), fibular nails (OPS codes: 5–790.3r, 5–790.4r, 5–790.5r, 5–793.ar, 5–793.br, 5–793.cr, 5–794.ar, 5–794.br, 5–794.cr). Based on these data sets, the treatment trends over the 15-year period as well as a possible influence of age or gender was investigated.

### Statistics

Next to descriptive statistics, a linear regression analysis was performed to analyze the incidence and age distribution. A Chi-squared-test was conducted for analysis of treatment strategies. Values are presented as mean ± standard deviation if not stated differently. All statistics were performed with SPSS Statistics (V27.0, IBM, Armonk, NY, USA). In all cases, significance was set at *p* < 0.05.

## Results

**Fig. 1:**
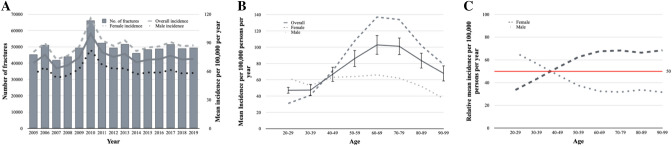
**A** Annual incidence. Bars: Absolute number of distal fibula fractures per year from 2005 to 2019, scale left side; lines: mean incidence per year per 100,000 persons separated for gender, scale on the right side. **B** Age distribution. Average mean incidence of distal fibula fractures per 100,000 persons per year separated for gender from 2005 to 2019. **C** Gender gap. Average relative mean incidence per 100,000 persons per year from 2005 to 2019

Between 2005 and 2019 a total of 745,823 ankle fractures with an involvement of the lateral malleolus were treated surgically in Germany. On average, 49,721 ± 5,464 (range 41,925–66,254) fractures were treated surgically per year (Fig. 1A). The patients’ mean age was 56.1 years (female: 60.5 years; male: 50.4 years) and 60.1% ± 0.6% (range 59.2–61.0%) of fractures occurred in females. The mean incidence per 100,000 inhabitants per year was 74 ± 32 (range 63.3–99.6) with no significant differences between 2005 and 2019 (*p* = *0.436*).

Figure 1B illustrates the mean incidence per 100,000 inhabitants per year according to the different age groups. The overall mean incidence revealed a peek between 60 and 80 years. Whereas the incidence for male patients showed no considerable age difference, the incidence for female patients did show a significant major increase between 40 and 70 years (*R*^2^ = 0.943, *p* = 0.006). This age–gender dependency is further highlighted in Fig. 1C. Whereas 66% of ankle fractures in patients aged 20–30 years occurred in males, approximately 70% of fractures in patients aged more than 60 years occurred in females.

**Fig. 2:**
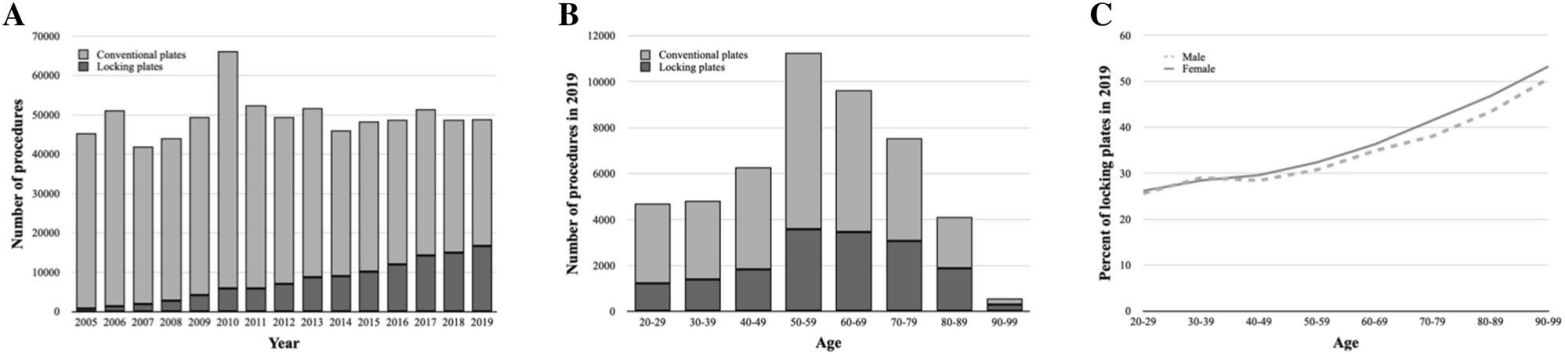
Locking plates vs. conventional plates. **A** Annual trends of utilized plate osteosynthesis from 2005 to 2019. **B** Age-dependent distribution in 2019. **C** Gender distribution in age-dependent ratio of angular stable locking plates in 2019

Figure 2 illustrates the treatment trends for non-locking conventional vs. locking plates. For the observed 15-year time period, the proportion of locking plates, compared to conventional plates, increased significantly from 2% in 2005 to 34% in 2019 (*p* < 0.0001; Fig. 2A). For 2019, the patients’ age showed a high correlation to the relative frequency of locking plates (*R*^2^ = 0.937, *p* < 0.001; Fig. 2B). Interestingly, no relevant gender difference could be observed for the application of locking plates (*p* = 0.712; Fig. 2C).

**Fig. 3:**
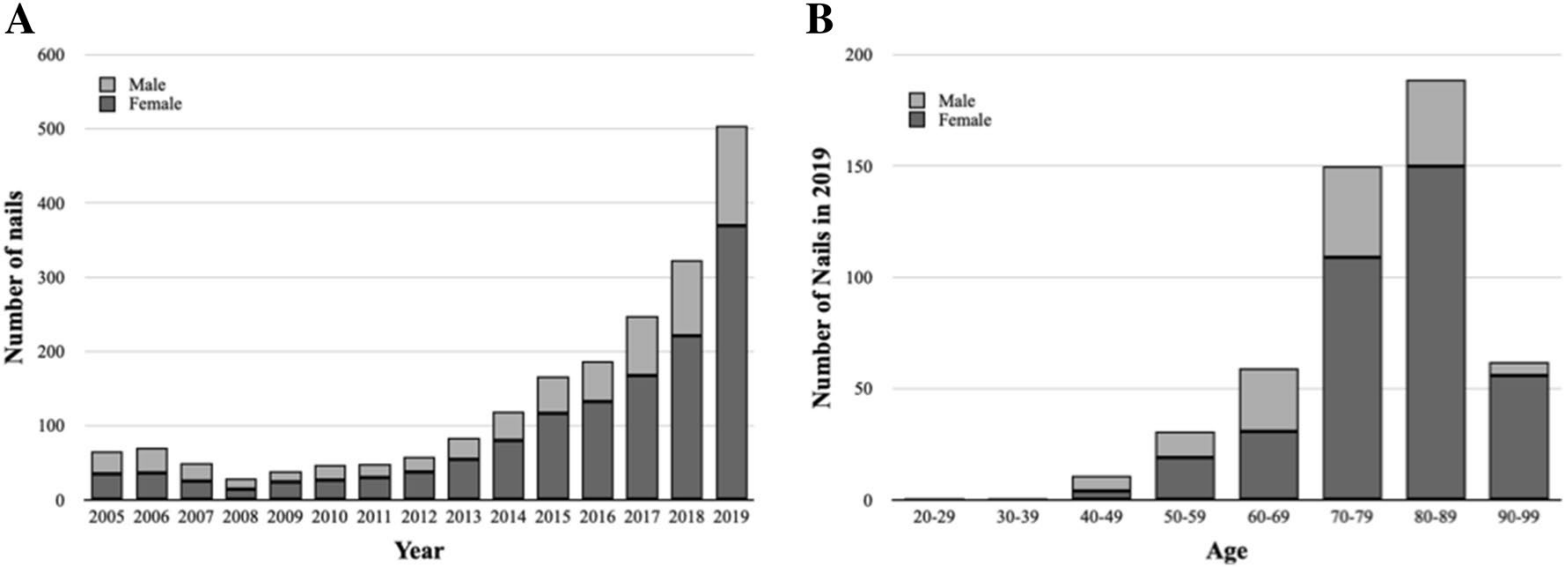
Intramedullary nails for distal fibula fractures. **A** Absolute annual number per year from 2005 to 2019, separated for gender. **B** Age-dependent distribution in 2019

Figure 3 illustrates the treatment trends for intramedullary fibular nails. The proportion of fibular nails overall increased significantly from 0.1% in 2005 to 1.0% in 2019 (*R*^2^ = 0.658, *p* < 0.001; Fig. 3A). Based on the 2019 data, there was a significant positive correlation between patients’ age and the relative frequency of intramedullary nails (*R*^2^ = 0.664, *p* = 0.014, see Fig. 3B). Contrary to the locking plates, intramedullary nails were more often used in female patients, increasing significantly with age to a peak of 89% female nails in the age group 90–99 years (R^2^ = 0.976, *p* < 0.001; Fig. 3B).

## Discussion

The analysis of national registries do not only allow to calculate precise epidemiological data, but also give an unbiased inside into the treatment reality and trends. The herein analyzed OPS data sets of the German Federal Statistical Office revealed a rather constant incidence for operatively treated ankle fractures involving the lateral malleolus over the past 15 years. Incidence rates varied considerably per age and sex. Whereas 66% of all fractures occurred in male patients in the age group 20–30 years, approximately 70% of all fractures occurred in female patients in the age group 60 years and above. Over the observed time period the proportion of locking plates for the distal fibula increased 17-fold and for fibular nails tenfold.

As outlined in the introduction, published incidence rates range considerably from 42 to 187/100,000 people per year [2–7, 22]. In contrast to the previous studies, we were only able to analyze the incidence of operatively treated ankle fractures with a fracture to the lateral malleolus. However, the threshold in Germany is very low for a surgical treatment decision, especially in younger patients [23]. The herein reported mean incidence rate of 74 ± 32 (range 63.3–99.6) surgically treated fractures per 100,000 inhabitants is, therefore, in the range of previously published studies. Interestingly, we found no significant difference in the incidence of operatively treated ankle fractures for the observed 15-year period. Kannus et al*.* described a massive increase of the incidence of ankle fractures in elderly patients from 57/100,000 people per year in 1970 to 150/100,000 people per year in 2000 [24]. However, the same authors showed in a follow-up study a steady incidence of 144/100,000 inhabitants per year between 1997 and 2004 [9]. A regional registry study by Elsoe et al. [4] demonstrated a constant incidence of ankle fractures in Denmark for a 10-year period between 2005 and 2014 with an incidence of 168.7/100,000 inhabitants per year [4].

Similar to previous studies, the herein presented study revealed an effect of gender and age to the incidence rates. Scheer et al*.* [2] analyzed the US National Electronic Injury Surveillance System from 2012 to 2016, which records any patients presenting to a US hospital emergency department. They reported an incidence of 42.2/100,000 people with an overall female dominance of 56% [2]. In line with our results, they described an age-dependent shift of the gender distribution with a male dominance < 30 years and an increasing relative female incidence of up to 70% for people older than 80 years [2]. Juto et al*.* [3] analyzed data from any hospital in Norrbotten County in Sweden from 2009 to 2013 and found an annual incidence of 179/100,000 persons, again with a higher percentage of female patients (58.4%) [3]. Again, the fracture incidence for female patients increased at the age of 30, whereas that of male patients showed a homogeneous distribution across all age groups. Tarantino et al*.* [8] conducted a 3-year study analyzing the hospitalization rate for various fractures, including ankle fractures, in people 45 years or above at 10 major Italian emergency departments. They reported a three times higher hospitalization rate for female patients above an age of 65 years [8]. In a prospective study of almost 8.000 people above the age of 55 years, Schuit et al*.* observed an almost four times higher incidence for female ankle fracture than for mens’ [25]. Consequently, the literature points, in line with our data, to a strong age–gender dependency of ankle fractures. The high percentage of male patients in the below 30-year groups could well be explained by risk behavior. The high female incidence for age groups above 40 years might indicate an association of ankle fractures to age and gender dependent bone quality impairment. Up to now, ankle fractures are neither considered fragility fractures nor commonly related to osteoporosis as no study has been able to proof a correlation between ankle fractures and a reduced bone mineral density [10, 26–28]. However, previous studies reported microarchitectural alterations on CT scans [29] and a risk increase for subsequent osteoporotic fractures for postmenopausal women with an ankle fracture compared to women without a fracture history [30–32].

This age-related impaired bone quality [10, 27, 28, 33] alongside an age dependent risk for complications [34] led to the development of novel treatment strategies, such as angular stable plates and fibular nails. Interestingly, specific prevalence studies on trends in treatment strategies for distal fibula fractures are spare. Today, it would be inconceivable not to treat a distal radius fracture or proximal humerus fracture with an angular stable implant. Therefore, it appears astonishing, that angular stable plates have not similarly become the standard of care for distal fibular fractures. Even more so, when considering that patients above the age of 75 years are unable to conduct partial weight-bearing [35]. Various authors have reported an increased use of locking plates at individual centers [36, 37], but comprehensive registry data are missing. The current study revealed a + 2115% increase over the observed 15-year time period in a major industrialized country. Controversy, we are still missing convincing evidence, that locking plates do decrease the complication risks compared to conventional plating [14, 38] and, therefore, outweigh the higher economic costs of locking plates compared to conventional plates [38].

Intramedullary nails for distal fibular fractures bear the advantage of reducing the invasiveness of the procedure at the cost of the quality of reduction. However, previous studies demonstrated not only fewer complications of intramedullary nails compared to standard plate osteosynthesis, but also an equal functional outcome [39, 40]. Despite an increase in the prevalence especially since 2012, the present study revealed a striking low quantity of 504 (1.01% of all osteosynthesis procedures) intramedullary nails used in distal fibula fractures in 2019. Scott et al*.* demonstrated a prevalence of intramedullary fixation for ankle fractures up to 20% in a recent british long-term register analysis [22]

There are certainly limitations to this study. The OPS data are a nationwide comprehensive data set, but as they provide data only on operatively treated patients, one cannot conclude the true incidence of these injuries, but rather the incidence of the performed surgeries. However, in Germany, there is a traditional low threshold for a surgical indication, especially in younger patients [23]. Therefore, the herein presented data rather underestimated the observed age-dependent incidence relation. Furthermore, the general incidence of 74 per 100,000 people per year is in line with a previous study from Thur et al*.* [5], who showed in a comparable nationwide analysis in Sweden an incidence of 71 ankle fractures per 100,000 inhabitants per year. The OPS codes themselves have limitations. They are directly linked to the case value and are a simplification of the actual fracture pattern. For example, multifragmentary fractures often have a higher case value than simple fractures and the OPS code does not allow to differentiate between medial and posterior malleolus fractures. To control for these OPS-inherent limitations, the authors focused on distal fibular fractures only and cumulated the corresponding OPS-codes. However, previous studies showed that in 75–90% of ankle fractures a fracture to the distal fibula was present [4, 7, 41, 42]. Finally, as the data are anonymized by the German Federal Statistical Office except for sex and age, one cannot conclude by the OPS code to the actual type of ankle fracture. Therefore, a possible influence of injury severity and the osteosynthesis strategy cannot be drawn. Despite these limitations, the herein analyzed data set is a complete representation of the treatment strategy in the most populous country in Europe.

In conclusion, the herein presented study is the first to present the incidence and treatment strategies for operatively treated distal fibula fractures between 2005 and 2019. Based on national registry data, a constant incidence rate was found with a gender and age dependent distribution. The treatment strategy over the observed 15-year period showed a steep increase for locking plates and intramedullary nails.

## Supplementary Information

Below is the link to the electronic supplementary material.

Supplementary file1 (DOCX 19 kb)
