# Supplementary material for: Trends of incidence and treatment strategies for operatively treated distal fibula fractures from 2005 to 2019: a nationwide register analysis
Source: Arch Orthop Trauma Surg. 2021 Nov 7;142(12):3771–7. doi: 10.1007/s00402-021-04232-0 (PMC9596585; doi:10.1007/s00402-021-04232-0)
Supplement: Supplementary file 1 — Supplementary file1 (DOCX 19 kb) [file 402_2021_4232_MOESM1_ESM.docx]

**SUPPLEMENTARY MATERIAL**

| OPS Code | Procedure |
| --- | --- |
| **5-790** | **Closed Reduction and Osteosynthesis** |
| 5-790.3r | Closed Reduction - Reamed intramedullary Nail |
| 5-790.4r | Closed Reduction - Locked intramedullary Nail |
| 5-790.5r | Closed Reduction - Intramedullary Nail with joint components |
| **5-793** | **Open Reduction, Simple Fracture** |
| 5-793.3r | Open Reduction - Simple Fracture – Conventional Plate |
| 5-793.ar | Open Reduction - Simple Fracture – Intramedullary Nail with joint component |
| 5-793.br | Open Reduction - Simple Fracture – Intramedullary Nail |
| 5-793.cr | Open Reduction - Simple Fracture – Intramedullary Nail with Transfixation |
| 5-793.kr | Open Reduction - Simple Fracture – Angular Stable Locking Plate |
| **5-794** | **Open Reduction, Multifragmentary Fracture** |
| 5-794.2 | Open Reduction – Multifragmentary Fracture – Conventional Plate |
| 5-794.ar | Open Reduction - Multifragmentary Fracture – Intramedullary Nail with joint component |
| 5-794.br | Open Reduction - Multifragmentary Fracture – Intramedullary Nail |
| 5-794.cr | Open Reduction - Multifragmentary Fracture – Intramedullary Nail with Transfixation |
| 5-794.kr | Open Reduction - Multifragmentary Fracture – Angular Stable Locking Plate |

Supplementary Table 1 List of included OPS codes in the study and their specific linked surgery.The final letter of the code points out to the anatomic region, all codes in this study are specific for “distal fibula” (letter “r”).
